# Tissue-Specific Impact of Autophagy Genes on the Ubiquitin–Proteasome System in *C. elegans*

**DOI:** 10.3390/cells9081858

**Published:** 2020-08-08

**Authors:** Sweta Jha, Carina I. Holmberg

**Affiliations:** Medicum, Department of Biochemistry and Developmental Biology, Faculty of Medicine, University of Helsinki, Haartmaninkatu 8, 00290 Helsinki, Finland; sweta.jha@helsinki.fi

**Keywords:** autophagy, ubiquitin–proteasome system, crosstalk, tissue specificity, *C. elegans*

## Abstract

The ubiquitin–proteasome system (UPS) and the autophagy–lysosomal pathway (ALP) are the two main eukaryotic intracellular proteolytic systems involved in maintaining proteostasis. Several studies have reported on the interplay between the UPS and ALP, however it remains largely unknown how compromised autophagy affects UPS function in vivo. Here, we have studied the crosstalk between the UPS and ALP by investigating the tissue-specific effect of autophagy genes on the UPS at an organismal level. Using transgenic *Caenorhabditis elegans* expressing fluorescent UPS reporters, we show that the downregulation of the autophagy genes *lgg-1* and *lgg-2 (ATG8/LC3/GABARAP)*, *bec-1 (BECLIN1)*, *atg-7 (ATG7)* and *epg-5* (mEPG5) by RNAi decreases proteasomal degradation, concomitant with the accumulation of polyubiquitinated proteasomal substrates in a tissue-specific manner. For some of these genes, the changes in proteasomal degradation occur without a detectable alteration in proteasome tissue expression levels. In addition, the *lgg-1* RNAi-induced reduction in proteasome activity in intestinal cells is not dependent on *sqst-1*/p62 accumulation. Our results illustrate that compromised autophagy can affect UPS in a tissue-specific manner, and demonstrate that UPS does not function as a direct compensatory mechanism in an animal. Further, a more profound understanding of the multilayered crosstalk between UPS and ALP can facilitate the development of therapeutic options for various disorders linked to dysfunction in proteostasis.

## 1. Introduction

Protein homeostasis (proteostasis) is a dynamic balance between protein biogenesis and degradation, and is essential for cell survival and growth. The pool of intracellular and various extracellular proteins is constantly being replaced by newly synthesized proteins, and therefore protein degradation must be selective and tightly regulated. In eukaryotes, the ubiquitin–proteasome system (UPS) and autophagy–lysosome pathway (ALP) are the two major intracellular proteolytic systems that mediate protein turnover. 

UPS is the main pathway responsible for the degradation of soluble and short-lived misfolded proteins, both in the cytosol and the nucleus. Proteasomal substrates are first polyubiquitinated via the action of three classes of enzymes: ubiquitin-activating enzymes (E1), ubiquitin-conjugating enzymes (E2) and ubiquitin ligases (E3), reviewed in [1,2]. The substrates are then degraded by the evolutionarily conserved 26S proteasome, which consists of a central barrel-shaped core particle (CP or 20S proteasome) enclosing the peptide hydrolysis activity of the complex. The 20S comprises four stacked heteroheptameric rings: two rings of α subunits at each end and two middle β subunit rings harboring the proteolytic activities. The 20S core particle is capped by one or two 19S regulatory particles (RP), required for substrate recognition, the removal of the attached ubiquitin chains, the ATP-dependent unfolding of the substrate, and transfer into the core for proteolysis, as reviewed in [3,4,5].

Long-lived misfolded proteins and defective cellular organelles are in turn degraded by the ALP. There are three main types of autophagy reviewed in [6], including macroautophagy, hereafter referred to as autophagy. The initiation of autophagy starts by the formation of an isolation membrane, the phagophore, which elongates to engulf the substrate(s), thus forming the double-layered autophagosome. This then fuses with late endosomes and lysosomes, leading to the formation of autolysosomes, where the substrate(s) is degraded by lysosomal hydrolases [2,7,8]. Autophagy is mediated through the conserved action of several proteins, from yeast to mammalian cells. A class-III phosphatidylinositol 3-kinase (PI3K) complex, together with lipid kinase VPS34 and the regulatory protein Beclin1, initiate the autophagic process by mediating vesicle nucleation. Two ubiquitin-like conjugation systems, encoded by the autophagy-related genes (atg), then regulate the elongation of the isolation membrane. The first conjugation system comprises ATG-12 and ATG-5, conjugated via a covalent bond mediated by the E1-like ATG-7 and E2-like ATG-10. The second conjugation system involves ATG-8, which conjugates with lipid phosphatidylethanolamine in a process regulated by ATG-7 and the E3-like ATG-3. The ATG-5-ATG-12 conjugate is detached after autophagosome formation, whereas ATG-8 remains in the autophagosome in its conjugated form. Currently, yeast ATG-8 and its mammalian homolog LC3 are the most commonly used markers for autophagic activity [9,10,11,12]. Another set of proteins functions at the last fusion step. RAB-7, a member of the Rab GTPase family, is present in late endosomes, and is involved in regulating the fusion to late lysosomes [12]. Additionally, mEPG-5 is required for the maturation of autophagosomes and their fusion to lysosomes [13,14]. The activation of autophagy can be induced by several stimuli, such as amino-acid deprivation, starvation or various stress conditions [15,16]. 

The UPS and ALP were previously believed to have exceedingly distinct substrates, and they were regarded as independent proteolytic systems, but accumulating evidence has established extensive crosstalk between the two, reviewed in [17]. Several studies using human cell lines and mice have reported that pharmacological and genetic inhibition of the UPS leads to the activation of autophagy [18,19,20,21]. However, the blocking of autophagy has reportedly more complex and contrasting results, both inhibiting and activating UPS [22,23,24,25]. 

Both the autophagy pathway and the UPS are highly conserved in the nematode *Caenorhabditis elegans*, reviewed in [26,27,28]. It has been reported that the rate of autophagic flux can vary in different *C. elegans* tissues during aging and stress [29,30]. Similarly, we have previously shown that UPS activity varies in a tissue-specific manner in *C. elegans* [31,32,33]. Here, we have investigated the crosstalk between UPS and ALP at an organismal level using *C. elegans.* We reveal that the downregulation of certain autophagy genes that function at different ALP steps elicits distinct tissue-specific effects on UPS in *C. elegans*, and that the observed changes in proteasomal degradation can occur either concomitant with, or without, a detectable change in proteasome tissue expression levels. 

## 2. Material and Methods

### 2.1. C. elegans and Growth Conditions

*C. elegans* strains were grown and maintained under standard conditions at 20 °C as described previously [34]. N2 (Bristol) and MAH215 strains were obtained from the Caenorhabditis Genetics Center (CGC). NL2099[*rrf-3(pk1426)II*] mutant strain was a kind gift from Dr. G. Wong (University of Eastern Finland). YD116[*rrf-3(pk1426);xzIs2[unc-54p::UIM2::ZsProSensor]*] reporter strain was generated by crossing NL2099[*rrf-3(pk1426)II*] strain with YD114[*xzIs2[unc-54p::UIM2::ZsProSensor]*] [35]. Information on the cloning of the plasmid has been described previously in Matilainen et al., 2013, as a precursor for the YD90[*xzIs1[vha-6p::UIM2::ZsProSensor]*] strain [32]. All strains used in this study are listed in Table S1.

### 2.2. C. elegans RNA Interference (RNAi)

RNAi was performed using the previously described feeding protocol [36]. The HT115 bacterial strain carrying the empty *pL4440* expression vector was used as a control in all experiments. Double stranded RNA expression was induced by adding 0.4 mM of isopropyl-β-D-thiogalactopyranoside (I6758, Sigma, St. Louis, MO, USA) during peak culture growth and its concentration was further increased to 0.8 mM just prior to the seeding of the feeding plates. Unless otherwise indicated, age-synchronized L1 larvae (day 1) were placed on control and RNAi-seeded plates, targeting either *lgg-1*, *lgg-2*, *bec-1*, *atg-7*, *rab-7*, *epg-5* or *sqst-1* (*C32D5.9*, *ZK593.6*, *T19E7.3*, *M7.5*, *W03C9.3*, *C56C10.12* or *T12G3.1*, respectively, Source BioScience, J. Ahringer library). Double RNAi of *lgg-1* and *sqst-1* was performed by mixing *lgg-1* and *sqst-1* RNAi cultures in 1:1 ratio according to their optical density prior to seeding. 

### 2.3. Microscopy and Image Analysis

Age-synchronized animals were imaged at first day of adulthood (day 4). Groups of animals were mounted on 3% agarose pad on glass slides and immobilized using 0.5 mM levamisole diluted in M9 buffer. The polyubiquitin reporter animals were imaged with a Zeiss Axio Imager upright epifluorescence microscope using 10× 0.3 NA EC Plan Neofluar objective. Images were quantified using the Fiji ImageJ software. 

For the UbG76V-Dendra2 and Dendra2 strains, images of a group of animals were taken before and immediately after proteins were converted from green to red fluorescence using 405 nm UV light (considered 0 h after conversion). Animals were recovered on corresponding feeding plates and reimaged after 6 h (intestinal reporter strains) or 24 h (body-wall muscle reporter strains). A Zeiss Axio Imager Z2 upright epifluorescence microscope was used for photoconversion and images were acquired with a 10 × 0.3 NA EC Plan Neofluar objective. Fluorescent intensities were quantified using the Fiji ImageJ software. Relative intensities were calculated by setting the absolute values of fluorescence intensity before and after photoconversion as 100%, for green and red fluorescence signals, respectively. For both the polyubiquitin reporter and the UPS activity reporter, images were exported into tiff-format and quantified using the original black and white version of the images without modifications. The background was subtracted using the corresponding command in Fiji software. The threshold was selected from the brightest image and this same threshold was applied to all images from the same experiment. The average of mean intensity was analyzed.

The autophagy dual marker reporter strain was imaged using Zeiss LSM880 confocal microscope (Motorized Zeiss Axio Observer Z1 inverted microscope). Z-stack images were acquired at 0.8 µm slice intervals with a 63× 1.4 NA plan-Apochromat objective. The z-stack images were converted to maximum intensity projection format using ZEN 2.1 (black version) and converted to tiff-format using Zen 2 lite (blue version). The number of puncta was calculated manually.

All images were processed with Adobe Photoshop CC 2018 software. When the brightness of an image was increased to make the fluorescent signal clearly visible, all corresponding images from the same experiment were modified in the same way. The number of animals imaged for each treatment and their *p*-values relative to the control are listed in Tables S2, S3 and S5 for the polyubiquitin reporter, UPS activity reporter and autophagy dual marker reporter, respectively.

### 2.4. Quantitative Real-Time PCR

Age-synchronized RNAi-treated animals were collected in M9 buffer at first day of adulthood (day 4) and stored at −80 °C. Total RNA was extracted using NucleoSpin RNA kit (Macherey-Nagel, Düren, Germany) and RNA concentration was measured with Nanodrop spectrophotometer at 260 nm. RT-PCR was performed using Maxima First Strand cDNA Synthesis Kit for RT-qPCR (Thermo Scientific, Waltham, MA, USA). The quantitative real-time PCR was done using Maxima SYBR Green/ROX qPCR Master Mix (2X) (Thermo Scientific) and LightCycler 480 (Roche, Basel, Switzerland) quantitative PCR machine. The data from qPCR were normalized to the geometric mean of mRNA concentration of three reference genes (*act-1*, *cdc-42* and *pmp-3*) [37]. The qPCR oligos used in this study are listed in Table S6.

### 2.5. In-Gel Proteasome Activity Assay and Western Blotting

RNAi-treated age-synchronized young adult (day 4) *rrf-3(pk1426)* animals were collected in M9 buffer prior to freezing at −80 °C for both the in-gel activity assay and Western blot samples. For in-gel activity assay, the animals were lysed in native gel lysis buffer using Dounce homogenizer as previously described [38]. The in-gel assay was performed as described earlier, but with slight modifications [32,38]. The native 3.5% acrylamide gels were run for 30 min at 20 mA and then for 2 h at 40 mA on an ice bath in a cold room (+4 °C). The gels were developed using developing buffer including 80 µM of the fluorogenic proteasome substrate suc-LLVY-AMC (I-1395, Bachem, Bubendorf, Switzerland). The gels were imaged with MultiImage Light Cabinet using FluorChem 8900 software (Alpha Innotech Corporation). After imaging, Coomassie staining of the gels was performed using Colloidal Blue Staining Kit (Invitrogen) to assess sample loading. The fluorescent signal was modified similarly for all images from the same experiment with Adobe Photoshop CC 2018 software, and signal intensities were quantified using Fiji ImageJ software. 

For Western blotting, the animals were lysed either according to the native gel protocol (detection of proteasome 20S alpha subunits) or by sonication using Western blot lysis buffer (50 mM Hepes (pH 7.4), 150 mM NaCl, 5 mM EDTA, 20 mM NEM, 10 µM MG-132 and protease inhibitor cocktail (Roche); detection of polyubiquitinated proteins). Samples were run on SDS-PAGE gel and immunoblotted onto a nitrocellulose membrane using Trans-Blot Turbo transfer system (Bio-Rad). Anti-20S alpha antibody (for proteasome 20S α-subunits 1–3 and 5–7, BML-PW8195, Enzo Life Sciences, 1:1000 dilution), FK-1 antibody (for polyubiquitinated proteins, BML-PW8805, Enzo Life Sciences, 1:500 dilution) and anti-α-tubulin antibody (for α-tubulin, T5168, Sigma, 1:10 000 dilution) were used for immunoblotting. The secondary antibodies for the proteasome 20S α-subunits and α-tubulin were anti-mouse IgG-HRP conjugates (W4021, Promega, 1:10,000 dilution), and anti-mouse IgM-HRP conjugates (401225, Calbiochem, 1:10,000 dilution) were used for polyubiquitinated proteins. Image Studio software (Licor) was used for imaging and quantifying the signals. A summary of the number of experiments and *p*-values relative to the respective controls is included in Table S4.

### 2.6. Immunohistochemical Analysis

Age-synchronized young adult (day 4) animals were collected in M9 buffer and fixed with 10% (*v*/*v*) phosphate buffered formalin as described earlier [33]. The fixed animals were embedded in 2% agar and the paraffin-embedded agar blocks were cut into 4 µm sections. Immunohistochemical staining was performed using Dako REAL^TM^ EnVision^TM^ Detection System, Peroxidase/DAB+, Rabbit/mouse kit (Dako) and the anti-20S alpha antibody (BML-PW8195, Enzo Life Sciences, 1:1000 dilution) [33]. The staining specificity of the anti-20S alpha antibody was previously established in *C. elegans* by omitting the primary antibody or by pre-absorption with purified 20S proteasome [33]. The stained slides were imaged with a Zeiss Axio Imager Z2 upright epifluorescence microscope using 10× 0.3 NA EC Plan Neofluar objective or 63× 1.4 NA Plan-Apochromat objective. Images were converted to tiff-format using Zen 2 lite (blue version). Immunostaining evaluation was performed independently and blindly by three investigators without prior knowledge of original treatment conditions. Staining intensity was scored as 0 for negative, 1 for mild, 2 for moderate and 3 for strong positive immunoreactivity. Information on the number of experiments and *p*-values relative to controls is presented in Table S4.

### 2.7. Statistical Analysis

Statistical significance was determined using the student’s *t*-test (two-tailed) in all quantifications and the treated group was compared to either control or to another treatment, as indicated in the figures.

## 3. Results

### 3.1. Downregulation of Autophagy Genes Affects the Accumulation of Polyubiquitinated Proteins in a Tissue-Specific Manner In Vivo

To investigate the interconnection between the UPS and autophagy at an organismal level, we downregulated the autophagy genes influencing different stages of ALP (Figure S1A) and analyzed the subsequent effects on the UPS in *C. elegans.* The RNAi-sensitive *rrf-3(pk1426)* strain was exposed to various RNAi treatments, targeting the individual autophagy genes *lgg-1*, *lgg-2* (homologs of mammalian LC3/GABARAP), *bec-1* (BECLIN 1), *atg-7* (ATG7), *rab-7* or *epg-5* (mEPG-5) (Figure S1B), resulting in a clear downregulation of their corresponding mRNA levels (Figure S1C). To monitor the outcome from the compromised autophagy gene expression, we used the dual fluorescence mCherry::GFP::LGG-1 reporter strain [29] to quantify the number of autophagosomes (APs) present in the live animals. As expected, RNAi against *lgg-1* or *bec-1* decreased the number of puncta that were positive for autophagosomes, whereas knockdown of *lgg-2*, *atg-7*, *rab-7* or *epg-5* significantly increased the formation of APs (Figure S2A, Table S5), validating our experimental setup.

**Figure 1:**
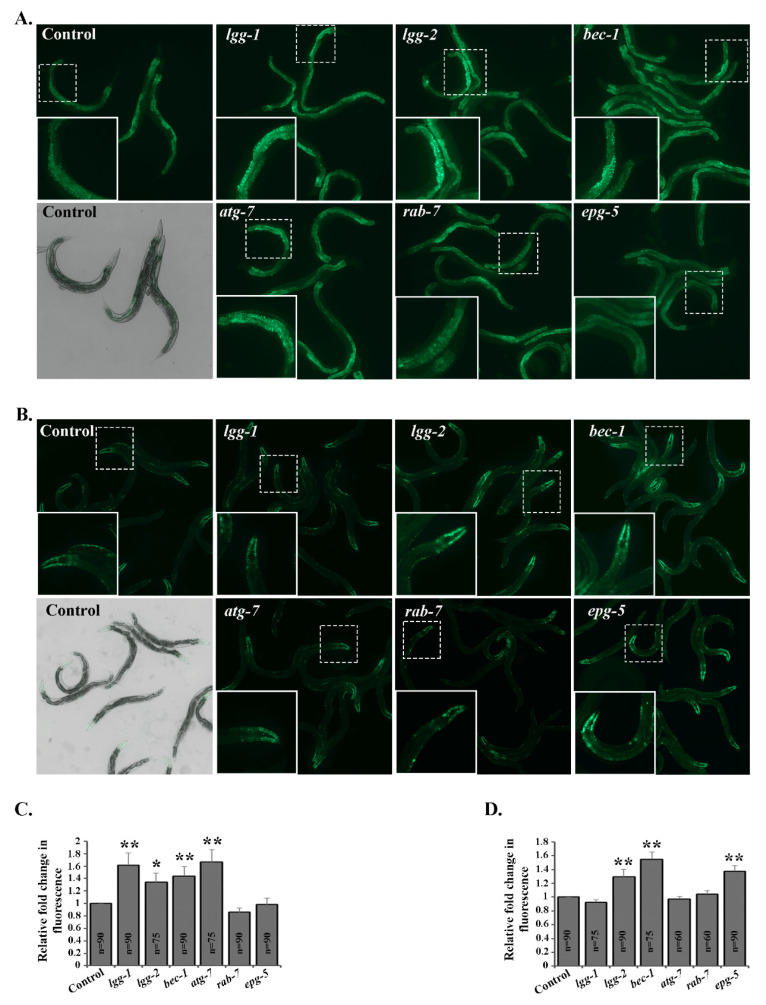
Downregulation of autophagy genes affects accumulation of fluorescent polyubiquitin reporters in a tissue-specific manner in vivo. Representative fluorescence micrographs of control, *lgg-1*, *lgg-2*, *bec-1*, *atg-7*, *rab-7* or *epg-5* RNAi-treated animals expressing the polyubiquitin reporter in the intestinal (**A**) and body-wall muscle (**B**) cells, respectively. Quantification of the fluorescent signal of the polyubiquitin reporter in intestinal (**C**) and body-wall muscle (**D**) cells, respectively. Graphs show average fold change in the amount of fluorescence signal compared to control RNAi (set as 1). Results are the mean of quantifications from 6 independent experiments (n = number of animals) (See Table S2). Error bars, SEM, * *p* < 0.05 and ** *p* < 0.01 compared to the control.

Next, we investigated the accumulation of polyubiquitinated proteins in live animals using our previously developed tissue-specific fluorescent polyubiquitin reporter, which binds to endogenous Lys-48-linked polyubiquitinated proteasomal substrates [32,39]. An increase in the fluorescence of the reporter corresponded with an accumulation of proteasomal substrates upon impaired proteasome function. Interestingly, animals expressing the polyubiquitin reporter in intestinal cells displayed increased accumulation of polyubiquitinated proteins upon introduction of RNAi against *lgg-1*, *lgg-2*, *bec-1* or *atg-7* (Figure 1A,C, Table S2). In contrast, the downregulation of *rab-7* or *epg-5* did not affect the reporter fluorescence in intestinal cells (Figure 1A,C). Additionally, we examined the effect of the knockdown of autophagy genes in animals expressing the polyubiquitin reporter in body-wall muscle cells.

Our results revealed that *lgg-2*, *bec-1* or *epg-5* RNAi increased the amounts of polyubiquitinated proteins in these cells, whereas *lgg-1*, *atg-7* or *rab-7* RNAi did not change the intensity of reporter fluorescence (Figure 1B,D, Table S2). When we investigated the total amounts of polyubiquitinated proteins present in whole animal lysates by Western blotting, we did not detect any differences between the control and RNAi-treated animals (Figure S3A,B). Taken together, our data indicate the tissue-specific effect of certain autophagy genes on the accumulation of polyubiquitinated proteasomal substrates.

### 3.2. Tissue-Specific Differences in UPS Activity In Vivo Upon Knockdown of Autophagy Genes

Next, we wanted to investigate whether the observed tissue-specific accumulation of polyubiquitinated proteins upon downregulation of autophagy genes could be a result of changes in proteasome activity. For this purpose, we took advantage of our previously generated photoconvertible UPS reporter system, which is based on the photoswitchable green-to-red fluorescent protein Dendra2 fused to the non-hydrolyzable ubiquitin moiety UbG76V [32,40]. The UbG76V-Dendra2 is targeted by the UPS, and degraded by the proteasome. After an UV light-induced photoconversion, the decrease in red fluorescence intensity of UbG76V-Dendra2 over time reflects UPS-mediated protein degradation, and in a manner independent of the translation of new reporter proteins. Here, we examined whether the modulation of autophagy gene expression directly affects the degradation of UbG76V-Dendra2 in different tissues. 

**Figure 2:**
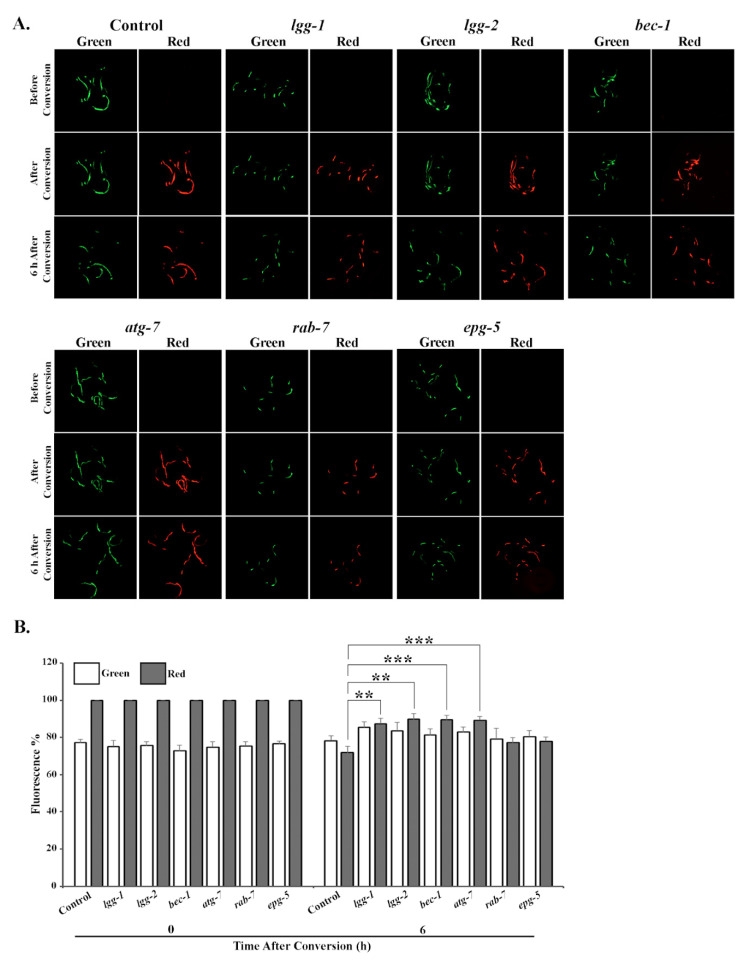
RNAi of *lgg-1*, *lgg-2*, *bec-1* or *atg-7* decreases UPS activity in intestinal cells in vivo. (**A**) The images represent fluorescent micrographs of control, *lgg-1*, *lgg-2*, *bec-1*, *atg-7*, *rab-7* or *epg-5* RNAi-treated N2 animals expressing UbG76V-Dendra2 reporter in intestinal cells before, immediately after and 6 h after photoconversion. (**B**) Quantification of UbG76V-Dendra2 degradation rate in intestinal cells. Graph shows the average percentage of green or red fluorescence relative to initial fluorescence intensity (set as 100%), or intensity at the point of photoconversion (set as 100%), respectively. The decrease in red fluorescence of UbG76V-Dendra2 reflects the UPS activity in the tissue. Results are the mean of quantification of 5 independent experiments (number of animals (n) = 105) (See Table S3). Error bars, SEM, ** *p* < 0.01 and *** *p* < 0.001 compared to the control.

**Figure 3:**
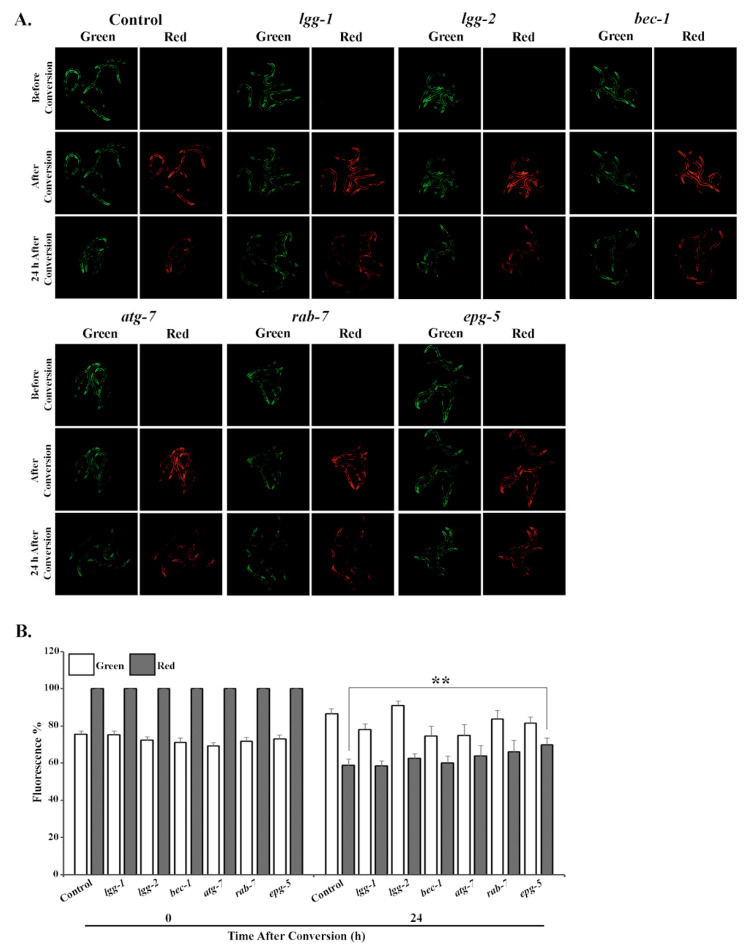
RNAi of *epg-5* decreases UPS activity in body-wall muscle cells in vivo. (**A**) The images represent fluorescent micrographs of control, *lgg-1*, *lgg-2*, *bec-1*, *atg-7*, *rab-7* or *epg-5* RNAi-treated wild-type animals expressing the UbG76V-Dendra2 reporter in body-wall muscle cells before, immediately after and 24 h after photoconversion. (**B**) Quantification of UbG76V-Dendra2 degradation rate in body-wall muscle cells. Graph shows the average percentage of green or red fluorescence relative to initial fluorescence intensity (set as 100%), or intensity at the point of photoconversion (set as 100%), respectively. The decrease in red fluorescence of UbG76V-Dendra2 reflects the UPS activity in the tissue. Results are the mean of quantification of 5 independent experiments (number of animals (n) = 100) (See Table S3). Error bars, SEM, ** *p* < 0.01 compared to the control.

Animals expressing UbG76V-Dendra2 in intestinal cells were fed either control, *lgg-1*, *lgg-2*, *bec-1*, *atg-7*, *rab-7* or *epg-5* RNAi bacteria, and analyzed for potential changes in reporter degradation. Six hours after photoconversion, approximately 10–12% of UbG76V-Dendra2 had been degraded in the *lgg-1*, *lgg-2*, *bec-1* or *atg-7* RNAi-treated animals, compared with the roughly 30% degradation rate observed in control animals (Figure 2A,B, Table S3). This slower degradation correlates with the increased accumulation of polyubiquitinated proteins (Figure 1A,C). In agreement with these results, RNAi against *rab-7* or *epg-5* did not affect UPS activity in intestinal cells (Figure 2A,B, Table S3). In contrast, the degradation rate of the UbG76V-Dendra2 reporter in body-wall muscle cells changed solely upon *epg-5* RNAi, resulting in approximately 15% difference compared with the control (Figure 3A,B, Table S3). Surprisingly, we did not detect a change in reporter degradation upon *lgg-2* and *bec-1* RNAi, although these treatments caused the accumulation of polyubiquitin reporter in the muscle cells. Further, the fluorescence in animals expressing the control Dendra2 in intestinal or body-wall muscle cells remained stable after RNAi against autophagy genes over the measured time period (Figures S4 and S5, Table S3). Altogether, our data demonstrate that the knockdown of specific autophagy genes induces distinct effects on UPS activity in intestinal and body-wall muscle cells in vivo.

### 3.3. Downregulation of lgg-1 Affects UPS Function Independently of p62/SQST-1 Accumulation

**Figure 4:**
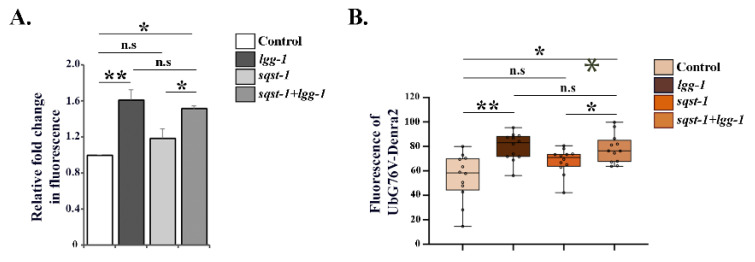
*lgg-1* RNAi affects UPS function independently of SQST-1. (**A**) Quantification of polyubiquitin reporter fluorescence in intestinal cells upon control, *lgg-1*, *sqst-1* or combined *sqst-1* and *lgg-1* RNAi. Graphs show average fold change in the amount of fluorescent signal compared to control RNAi treatment (set as 1). Results are the mean of quantifications of 4 independent experiments (number of animals (n) = 90). (**B**) Quantification of UbG76V-Dendra2 degradation rate in intestinal cells. Graph shows the average percentage of red fluorescence remaining 6 h after photoconversion upon respective RNAi treatments. Results are the mean of quantification of 3 independent experiments (number of animals (n) = 65) (See Tables S2 and S3). Error bars, SEM, * *p* < 0.05, ** *p* < 0.01, and n.s. = non-significant compared to control or indicated treatment.

Previous studies have reported that polyubiquitinated proteins accumulate in mammalian cells upon autophagy inhibition via a process mediated by increased levels of the autophagosome cargo protein p62 [23,41]. We performed RNAi against *sqst-1*, the *C. elegans* homolog of mammalian p62, in combination with *lgg-1* RNAi to prevent p62 accumulation. Simultaneous downregulation of these two genes did not counteract the *lgg-1* RNAi-induced accumulation of polyubiquitinated proteins, nor the reduction in proteasomal substrate degradation in intestinal cells (Figure 4A,B). This suggests that the decreased UPS activity observed upon *lgg-1* RNAi is independent of *sqst-1* upregulation, as *sqst-1* mRNA levels were efficiently decreased upon both single or double gene RNAi treatment (Figure S1D). *sqst-1* RNAi alone did not elicit an effect on proteasome activity in intestinal cells (Figure 4A,B). Our data imply that modulating autophagy via decreased *lgg-1* expression leads to impaired proteasome activity, in a SQST-1-independent manner, in *C. elegans* intestinal cells. 

### 3.4. Depletion of Autophagy Genes Affects Proteasome Activity or Expression

**Figure 5:**
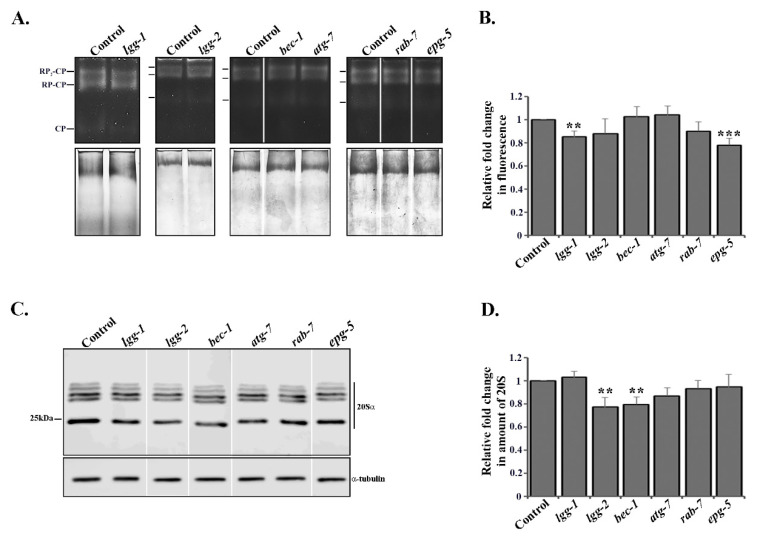
Autophagy genes affect proteasome activity or expression. (**A**) Proteasome activity investigated by in-gel activity assay with fluorogenic suc-LLVY-AMC proteasome substrate from whole animal lysates of *rrf-3(pk1426)* animals treated with control, *lgg-1*, *lgg-2*, *bec-1*, *atg-7*, *rab-7* or *epg-5* RNAi (upper panel), and Coomassie staining of total protein of the same gel (lower panel). The marker lines represent RP_2_-CP, RP-CP and CP, respectively. (**B**) Quantification of in-gel proteasome activity. The bands corresponding to RP_2_-CP, RP-CP and CP were used for quantification. Graph shows average fold change in fluorescent signal compared to control RNAi (set as 1). Results are the mean of quantifications from 10 independent experiments (See Table S4). Error bars, SEM, ** *p* < 0.01 and *** *p* < 0.001 compared to the control. (**C**) Lysates of *rrf-3(pk1426)* animals treated with control, *lgg-1*, *lgg-2*, *bec-1*, *atg-7*, *rab-7* or *epg-5* RNAi separated on SDS-PAGE and immunoblotted against proteasome 20S alpha subunits (upper panel) and α-tubulin (lower panel). (**D**) Quantification of proteasome immunoblots. Graph shows average fold change in levels of 20S alpha subunits normalized against α-tubulin. Results are the mean of quantifications from 7 independent experiments (See Table S4). Error bars, SEM, ** *p* < 0.01 compared to the control.

In addition to the UPS in vivo experiments, we investigated proteasome activity in whole animal lysates by performing an in-gel activity assay whereby the lysates were resolved on a native acrylamide gel, and the in-gel activity was measured with the fluorogenic suc-LLVY-AMC proteasome substrate [38]. Knockdown of *lgg-1* or *epg-5* by RNAi decreased proteasome activity by approximately 0.2-fold, compared to the control (Figure 5A,B, Table S4), which is in agreement with our in vivo data (Figure 2A,B and Figure 3A,B, Table S3). Interestingly, we did not detect any effect on proteasome activity after *lgg-2*, *bec-1* or *atg-7* RNAi treatments (Figure 5A,B, Table S4), suggesting that the in vivo tissue-specific proteasome activity responses (Figure 2 and Figure 3, Table S3) are masked in whole animal lysates.

We next investigated total proteasome amounts in whole worm lysates with Western blot, using an antibody against proteasome 20S alpha subunits. The downregulation of *lgg-2* or *bec-1* resulted in approximately a 0.2-fold decrease in total proteasome abundance, whereas no change could be detected with *lgg-1*, *atg-7*, *rab-7* or *epg-5* RNAi (Figure 5C,D, Table S4). 

Taken together, our in vivo and in vitro results reveal that the depletion of autophagy genes causes different proteasomal responses, i.e., *lgg-1* and *epg-5* RNAi modulate proteasome activity without detectably changing its level, whereas *lgg-2* and *bec-1* RNAi in turn decrease total proteasome amount in whole animal lysates without causing changes in proteasome activity.

### 3.5. RNAi of lgg-2 or bec-1 Causes Distinct Responses in Proteasome Tissue Expression

**Figure 6:**
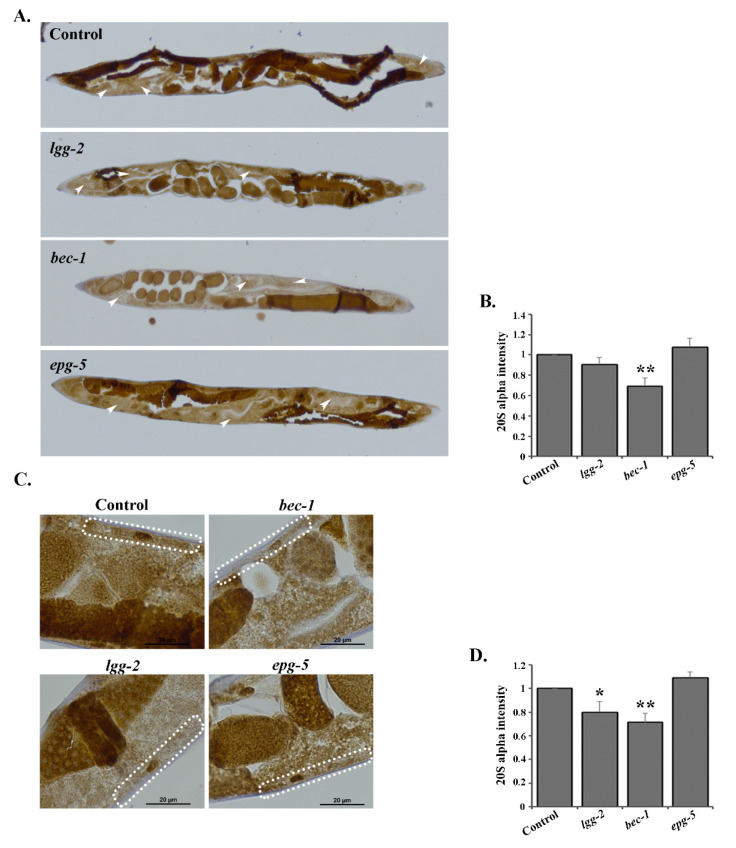
Knockdown of *lgg-2* and *bec-1* results in tissue-specific proteasome expression responses. (**A**) Images representing proteasome immunoreactivity of *rrf-3(pk1426)* animals treated with control, *lgg-2*, *bec-1* or *epg-5* RNAi in the intestinal cells (indicated by white arrowheads). (**B**) Quantification of immunoreactivity in intestinal cells. Graph shows the average fold change in staining intensity of 20S alpha antibody compared to control treatment (set as 1). Results are the mean of quantifications from 3 independent experiments (number of intestinal cells (n) = 80) (See Table S4). Error bars, SEM, ** *p* < 0.01 compared to the control. (**C**) Higher magnification images of *rrf-3(pk1426)* animals treated with control, *lgg-2*, *bec-1* or *epg-5* RNAi in the body-wall muscle cells (outlined with white dash line). (**D**) Quantification of immunoreactivity in the body-wall muscle cells. Graph shows the average fold change in staining intensity against 20S alpha antibody. Results are the mean of quantifications from 3 independent experiments (number of muscle cells (n) = 45) (See Table S4). Error bars, SEM, * *p* < 0.05, ** *p* < 0.01 compared to control.

To further investigate proteasome expression at the tissue and cell resolution level in *C. elegans*, we used an immunohistochemical approach for the detection of the 20S proteasome [33]. In support of our Western blot data from the whole animal lysates, we detected decreased proteasome amounts after the downregulation of *lgg-2* or *bec-1* by RNAi (Figure 6). Importantly, the decrease in proteasome expression was not uniform, but displayed distinct tissue-specificity. *bec-1* RNAi decreased proteasome amounts in both intestinal and body-wall muscle cells (approximately by 0.25-fold compared to control RNAi, Figure 6A–D, Table S4), whereas *lgg-2* RNAi caused a decrease in proteasome expression only in the body-wall muscle cells (approximately by 0.2-fold, Figure 6C,D, Table S4). In agreement with our Western blot analysis, no changes were detected with *epg-5*, *lgg-1*, *atg-7* or *rab-7* RNAi (Figure 6 and Figure S6, Table S4). Altogether, our studies reveal that the downregulation of various autophagy genes leads to distinct responses in proteasome tissue expression levels.

## 4. Discussion

In this study, we use *C. elegans* as an organismal approach to evaluate for the first time how compromising autophagy at different steps affects UPS in different tissues. We show that individual depletions of several autophagy genes cause tissue-specific effects on UPS activity and proteasome abundance. Though several previous studies using cell lines and mice have shown that the impairment of proteasome function results in the induction of autophagy [18,19,20,21,42,43], studies on the inhibition of autophagy have reported more complex results, including both UPS activation and inhibition. For example, the pharmacological inhibition of autophagy and the knockdown of *Atg-5* or *Atg-7* in human colon cancer cell lines have been shown to increase proteasomal activity [24]. Similarly, Kim et al. reported that *Atg-5* deficient mouse embryonic fibroblast exhibited elevated proteasome activity [22]. On the other hand, *Atg-7* deficient mouse neurons display accumulations of ubiquitinated substrates without observed changes in proteasomal function [44]. Additionally, Korolchuk et al. reported that arresting autophagy by siRNA knockdown of *Atg-7* and *Atg-12*, or with autophagy inhibitors, causes impaired degradation of at least two proteasomal substrates, UbG76V-GFP and p53, in HeLa cells, although without affecting total proteasome activity in the cell lysates [23]. Furthermore, autophagy blockage due to lysosomal enzymatic deficiency causes decreased proteasomal activity in neuroblastoma cells [25]. Here, we show that downregulation of *bec-1*, *atg-7*, *lgg-1*, *lgg-2* or *epg-5* impairs UPS function in *C. elegans*. Importantly, our results reveal that, depending on which autophagy gene is depleted, the effect on UPS varies. For example, downregulation of *lgg-1* or *epg-5* by RNAi affects the UPS and proteasomal activity in in vivo and in vitro assays, without a concomitant change in proteasome expression, whereas *lgg-2* or *bec-1* RNAi decreases UPS activity in vivo, as well as the amount of the proteasome (Figure 2, Figure 3, Figure 5 and Figure 6). The results of *lgg-1* and *epg-5* RNAi suggest that their effect could be mediated through a regulator of the proteasome. In line with this suggestion, we have previously shown that the proteasome-associated deubiquitinase UBH-4 regulates proteasome activity in *C. elegans* in an insulin/IGF-1 signaling-mediated manner [32], and that animals with mutations in this pathway display altered proteasome activity without a concomitant change in proteasome tissue expression [32,33]. Further studies are needed to uncover the underlying mechanisms of *lgg-1* and *epg-5* knockdown in proteasome activity. 

Our previous research has demonstrated that UPS activity varies in a tissue-specific manner in live *C. elegans*, and is affected by aging and stress, such as proteasomal impairment and elevated growth temperature [31,32,35]. The rate of autophagic flux has also been reported to vary in different *C. elegans* tissues [29,30]. Chang et al. showed that autophagy activity declines in several tissues in wild-type animals, whereas long-lived mutants display spatiotemporal regulation of autophagy [29]. Additionally, Chapin et al. reported that both basal autophagy as well as stress-induced autophagy can vary in different tissues [30]. Recently, it was shown that affecting proteostasis through the downregulation of chaperones involved in disaggregation results in increased autophagy and decreased UPS in *C. elegans* and human HEK293 cells [45]. Interestingly, the same study revealed that both autophagy and UPS are impaired in tissues expressing the human disease-linked amyloid protein Aβ_3–42_ and Q_40_ in *C. elegans* [45]. Here, we reveal that a widespread downregulation of distinct autophagy genes generates tissue-specific UPS responses. We show that the downregulation of *bec-1*, *lgg-1*, *lgg-2* and *atg-7* decreases UPS activity in intestinal cells, whereas *epg-5* RNAi affects UPS activity in muscle cells (Figure 2 and Figure 3). Similarly, we detect tissue-specific decreases in proteasome expression upon autophagy impairment, as *bec-1* RNAi decreases the amount of proteasome in both intestinal and muscle cells, but *lgg-2* RNAi affects proteasome expression only in muscle cells (Figure 6). This tissue specificity is not due to a difference in RNAi efficiency, as gene-specific RNAi phenotypes are observed for both cell types. It is noteworthy that our data suggest that a particular proteasomal substrate can be degraded while some substrates accumulate upon compromised autophagy.

Our experimental approaches for studying UPS activity and proteasome expression enable us to perform intra-tissue analysis at the organismal level. The *bec-1* RNAi-induced accumulations of the UPS substrate UbG76V-Dendra2, as well as those of the endogenous pool of K-48-linked polyubiquitinated proteins in the intestine, coincided with the decreased expression of proteasome in the same tissue, thus displaying a mechanism by which BEC-1 affects intestinal UPS function. The instances wherein we did not observe a correlation between changes in proteasome level and UPS activity (i.e., downregulation of *lgg-1*, *lgg-2*, *atg-7* and *epg-5*) could be due to, for example, changes in substrate delivery to the proteasome, posttranslational modifications, or proteasome interaction with regulators. Korolchuk et al. has demonstrated that, in HeLa cells, the accumulation of distinct proteasomal substrates upon compromised autophagy is dependent on increased p62 levels without a change in proteasome activity, suggesting difficulties in substrate delivery to the proteasome [23]. Here, we show that the accumulation of endogenous K-48-linked polyubiquitinated proteasomal substrates, as well as the decreased degradation of the proteasomal substrate UbG76V-Dendra2 upon impaired autophagy by *lgg-1* RNAi, is independent of increased levels of SQST-1 in *C. elegans* intestinal cells. As p62/SQST-1 has preference for binding to K-63-linked polyubiquitinated proteins [46], this may explain why the pool of endogenous K-48-linked proteasomal substrates is not affected. Moreover, it was recently shown that overexpression of *sqst-1* in *C. elegans* leads to a modest increase in proteasome activity, and a reduced amount of ubiquitinated proteins, as analyzed in whole animal lysates [47].

Our data demonstrate that UPS does not function as a direct compensatory mechanism upon ALP impairment in a multicellular organism. Importantly, our results emphasize the existence of tissue-specific responses in UPS activity and proteasome expression to disturbed autophagy. Whilst previous research has largely focused on how UPS and autophagy are individually regulated, our study provides new information that could be implemented for the simultaneous modulation of UPS and ALP. This is of particular interest, as dysfunctions in these proteolytic systems are associated with various, often aging-related, disorders, such as neurodegenerative diseases (e.g., Parkinson’s disease and Alzheimer’s disease), autoimmune diseases and different types of cancer. In particular, the tissue-specific effects of autophagy gene depletions on UPS revealed here could provide insight into designing therapies for altering proteasome activity in a specific tissue. Our results create further interest in discovering the mechanisms by which the depletion of different autophagy genes elicits distinct UPS responses. More detailed understanding of UPS and ALP, and their multifaceted crosstalk in varying physiological conditions, will increase our understanding of proteostasis regulation.

## Supplementary Materials

The following are available online at https://www.mdpi.com/2073-4409/9/8/1858/s1. Figure S1: Efficient downregulation of autophagy genes following RNAi. Figure S2: Expression of *mCherry::GFP::LGG-1* reporter in *C. elegans* following RNAi of autophagy genes. Figure S3: Downregulation of autophagy genes does not affect the amount of polyubiquitinated proteins in whole animal lysates. Figure S4: Knockdown of autophagy genes does not affect the stability of Dendra2 reporter in intestinal cells. Figure S5: Knockdown of autophagy genes does not affect the stability of Dendra2 reporter in body-wall muscle cells. Figure S6: RNAi of *lgg-1*, *atg-7* and *rab-7* does not affect proteasome tissue expression. Table S1: List of *C. elegans* strains used in this study. Table S2: Numbers of imaged polyubiquitin reporter animals. Table S3: Numbers of imaged UbG76V-Dendra2 and Dendra2 reporter animals. Table S4: Significance values of in vitro assays. Table S5: Numbers of imaged *mCherry::GFP::LGG1* reporter animals. Table S6: List of qPCR oligonucleotides used in this study.
