# Supplementary material for: Tissue-Specific Impact of Autophagy Genes on the Ubiquitin–Proteasome System in C. elegans
Source: Cells. 2020 Aug 8;9(8):1858. doi: 10.3390/cells9081858 (PMC7464313; doi:10.3390/cells9081858)
Supplement: Supplementary file 1 [file cells-09-01858-s001.pdf]

# Supplementary materials

A.

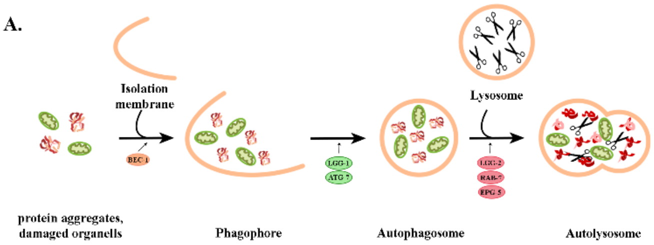


B.

| *C. elegans* genes | Mammalian genes | Yeast genes |
|---|---|---|
| *bec-1* | *BECLIN1* | *VPS30/ATG6* |
| *lgg-1* | *LC3/GABARAP* | *ATG-8* |
| *lgg-2* | *LC3/GABARAP* | *ATG-8* |
| *atg-7* | *ATG-7* | *ATG-7* |
| *rab-7* | *RAB-7* | - |
| *epg-5* | *mEPG-5* | - |

C.

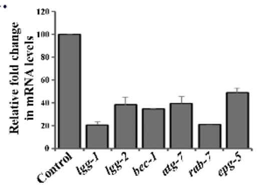


D.

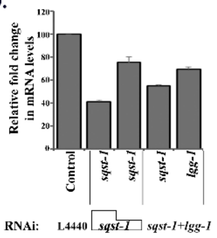


**Figure S1. Efficient downregulation of autophagy genes following RNAi** (A) Overview of the Autophagy Lysosomal Pathway (ALP). The pathway starts with the formation of an isolation membrane and ends with the fusion of the autophagosome with the lysosome. Autophagy genes investigated in this study are indicated according to their role at the respective steps in the pathway. (B) *C. elegans* genes used in this study and their homologs in human and yeast. (C) and (D) RNAi efficiently decreases mRNA levels of the corresponding genes. (C) Expression of *lgg-1, lgg-2, bec-1, atg-7, rab-7* or *epg-5* mRNA in RNAi-treated *rrf-3(pk1426)* animals was investigated with qPCR. Graph shows the average fold change in mRNA levels compared to control (set as 1). Results are mean of 2-3 independent experiments in triplicate, except for *bec-1* and *rab-7* RNAi with one experiment in triplicate. Error bar, SD. (D) Expression of *sqst-1* or *lgg-1* mRNA levels upon RNAi targeting with either empty vector control (L4440), *sqst-1,* diluted *sqst-1* (RNAi with *sqst-1* and L4440 in 1:1 ratio) or both *sqst-1* and *lgg-1* (RNAi of *sqst-1* and *lgg-1* in 1:1 ratio). Graph shows the average fold

change in mRNA levels compared to control (set as 1). Results are mean of 2-3 independent experiments in triplicate. Error bar, SD.

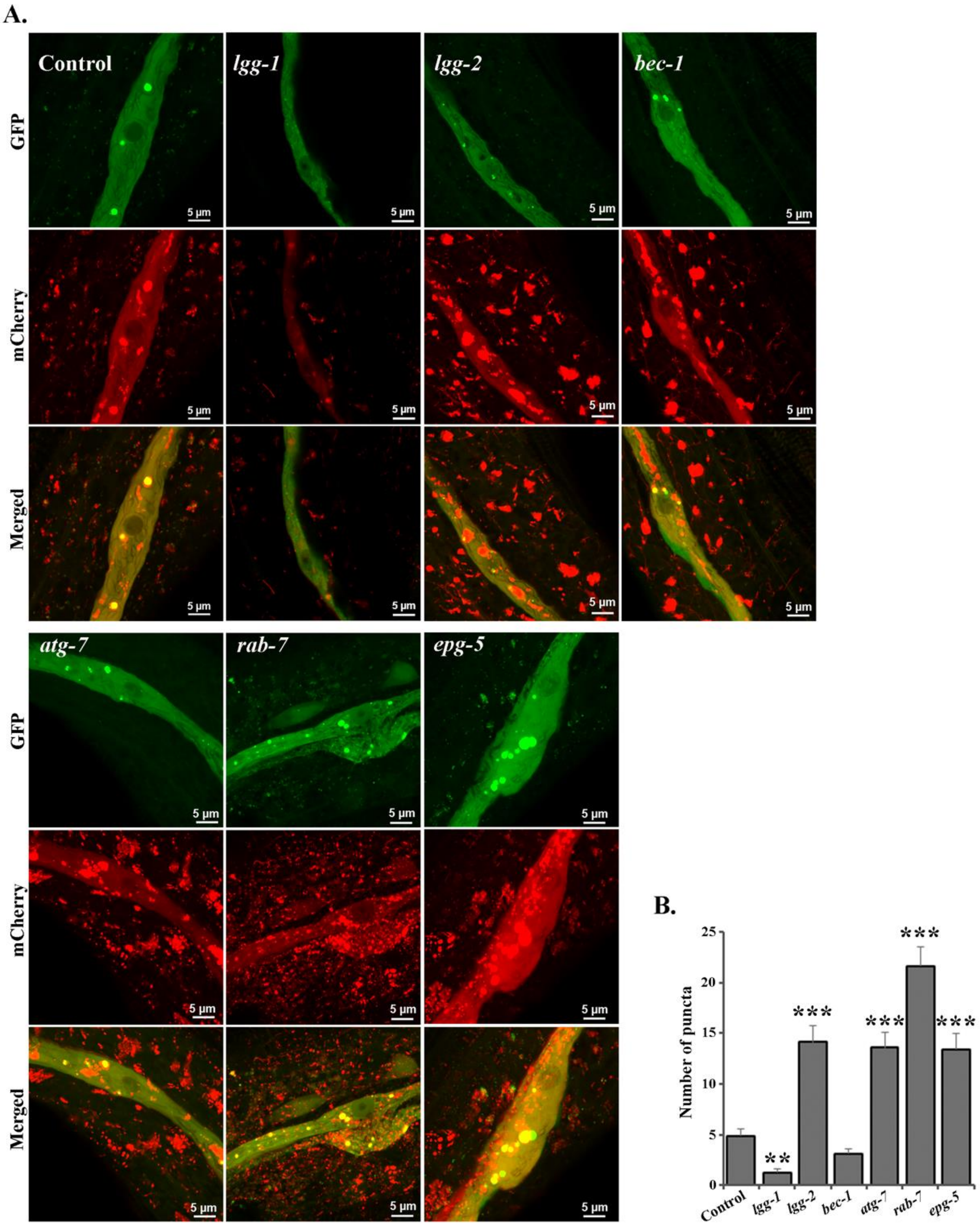


**Figure S2. Expression of *mCherry::GFP::LGG-1* reporter in *C. elegans* following RNAi of autophagy genes.** (A) Images representing fluorescent micrographs of transgenic N2 (wild-type) animals expressing *mCherry::GFP::LGG-1* after treatment with control, *lgg-1, lgg-2, bec-1, atg-7, rab-7* or *epg-5* RNAi. (B) Quantification of the number of autophagosomes (APs, overlayed GFP and mCherry positive puncta) in hypodermal seam cells. Results are the

mean of quantifications from 3 independent experiments (number of hypodermal cells = 12) (See Table S5). Error bars, SEM, $^{**}P<0.01$ and $^{***}P<0.001$ compared to control.

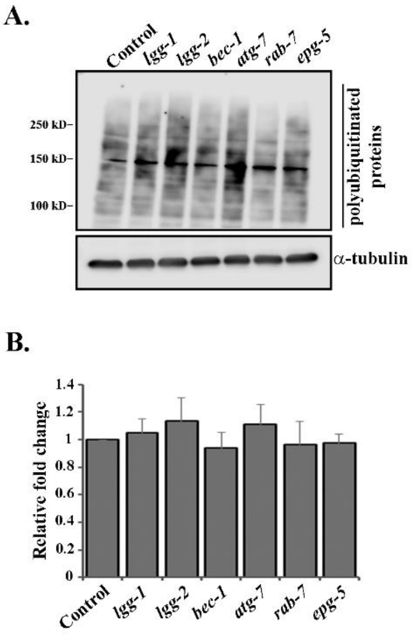


**Figure S3. Downregulation of autophagy genes does not affect the amount of polyubiquitinated proteins in whole animal lysates.** (A) Lysates of *rrf-3(pk1426)* animals treated with control, *lgg-1, lgg-2, bec-1, atg-7, rab-7* or *epg-5* RNAi were separated on SDS-PAGE and immunoblotted against polyubiquitinated proteins (upper panel) and α-tubulin (lower panel). (B) Quantification of the amount of polyubiquitinated proteins. Graph shows the average fold change in the amount of polyubiquitinated proteins compared to control RNAi (set as 1) and normalized against α-tubulin. Results are the mean of quantifications of 5 independent experiments. Error bars, SEM.

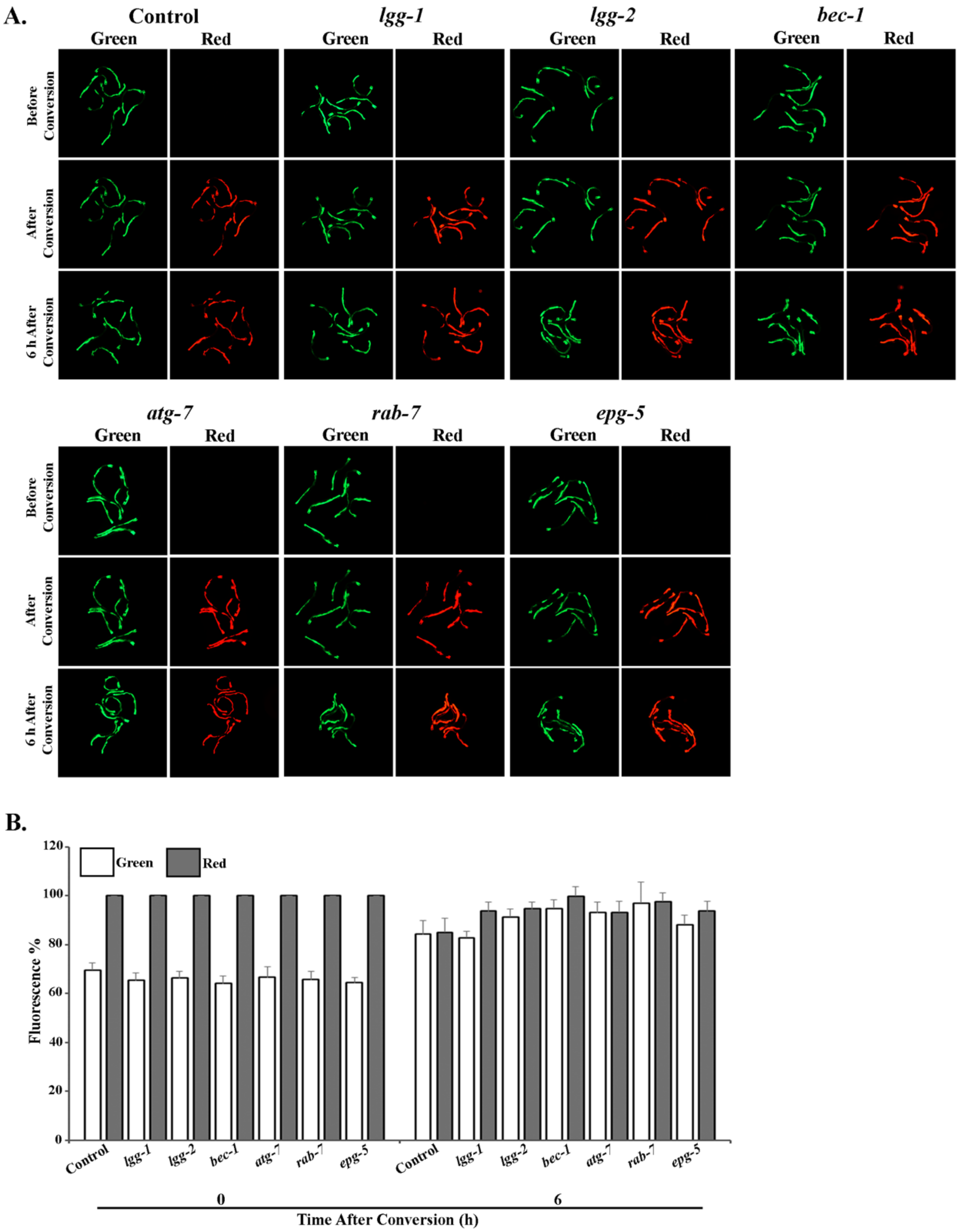


**Figure S4.** **Knockdown of autophagy genes does not affect the stability of Dendra2 reporter in intestinal cells.** (A) Fluorescent micrographs of control, *lgg-1, lgg-2, bec-1, atg-7, rab-7* or *epg-5* RNAi treated N2 animals expressing Dendra2 in intestinal cells before, immediately after, and 6 h after photoconversion. (B) Quantification of Dendra2 degradation

in intestinal cells. Graph shows the average percentage of green of red fluorescence relative to initial fluorescent intensity (set as 100%) or intensity at the point of photoconversion (set as 100%), respectively. Results are the mean of quantification from 3 independent experiments (number of animals (n) = 60) (See Table S3). Error bars, SEM.

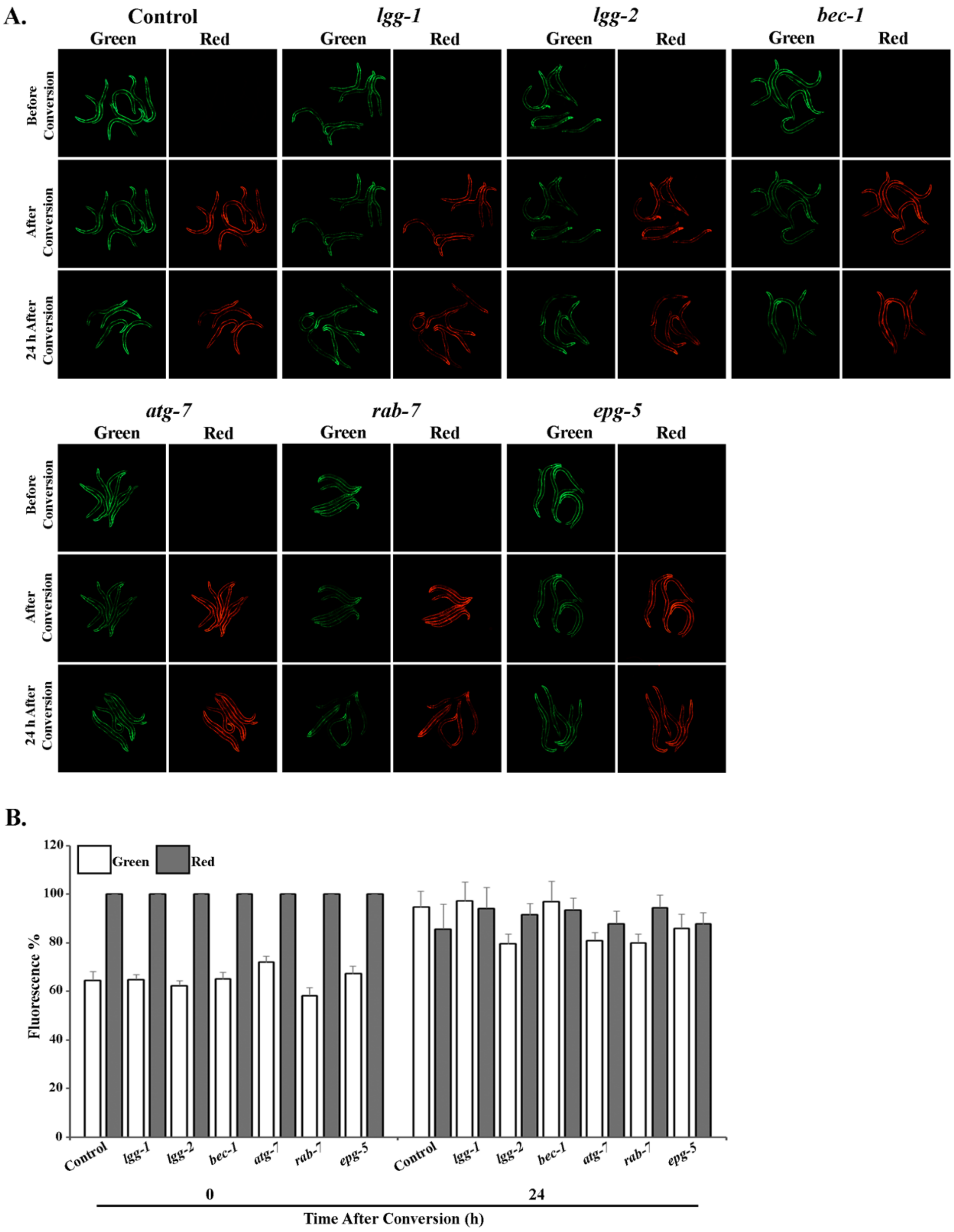


**Figure S5.** **Knockdown of autophagy genes does not affect the stability of Dendra2 reporter in body-wall muscle cells.** (A) Fluorescent micrographs of control, *lgg-1, lgg-2, bec-1, atg-7, rab-7* or *epg-5* RNAi treated N2 animals expressing Dendra2 in body-wall muscle cells before, immediately after, and 24 h after photoconversion. (B) Quantification of Dendra2

degradation in body-wall muscle cells. Graph shows the average percentage of green of red fluorescence relative to initial fluorescence intensity (set as 100%) or intensity at the point of photoconversion (set as 100%), respectively. Results are the mean of quantification from 3 independent experiments (number of animals (n) = 60) (See Table S3). Error bars, SEM. (Table S3)

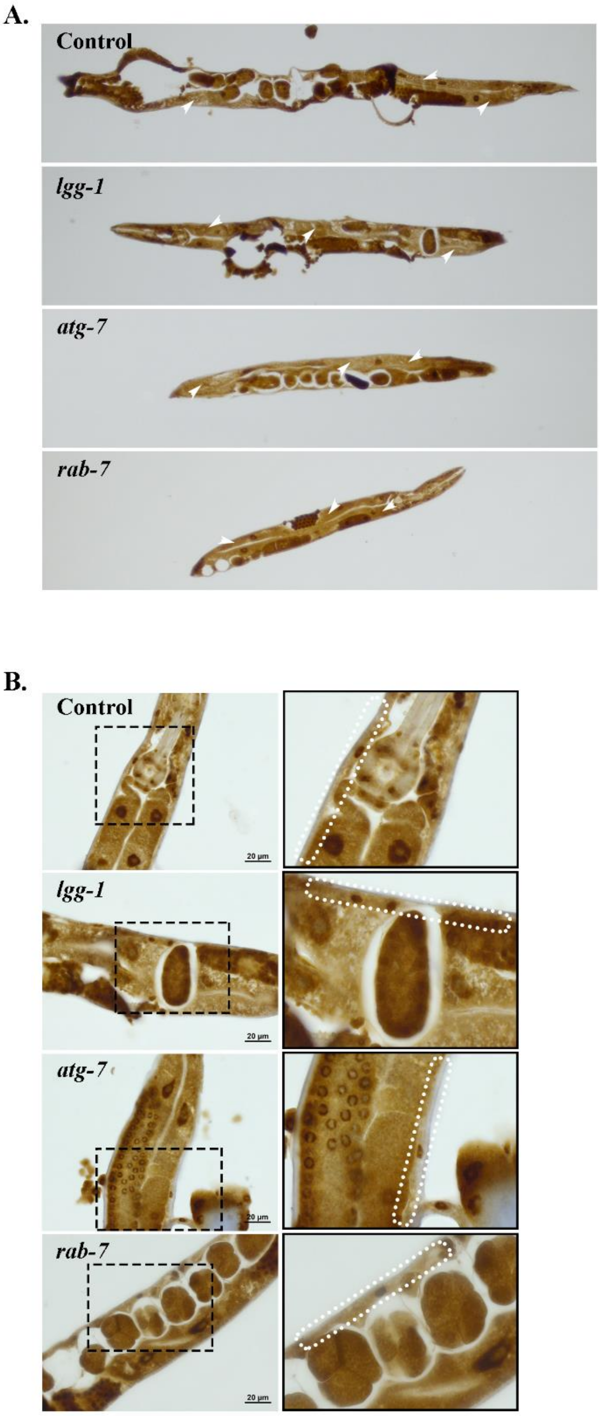


**Figure S6. RNAi of *lgg-1, atg-7* and *rab-7* does not affect proteasome tissue expression.** (A) Images showing 20S proteasome immunoreactivity of *rrf-3(pk1426)* animals treated with control, *lgg-1, atg-7* or *rab-7* RNAi in the intestinal cells (indicated by white

arrowheads). (B) Higher magnification images showing 20S immunoreactivity in body-wall muscle cells (outlined with white dash line).

**Table S1**. **List of *C. elegans* strains used in this study**

| Strain | Genotype | Origin |
|---|---|---|
| [1]N2 | | CGC |
| [2]NL2099 | *rrf-3(pk1426)* | CGC |
| [3]MAH215 | *sqIs11[lgg-1p::mCherry::gfp::lgg-1 + rol-6]* | CGC |
| [4]YD1 | *xzEx1[Punc-54::Dendra2]* | [40] |
| [5]YD3 | *xzEx3[Punc-54::UbG76V::Dendra2]* | [40] |
| [6]YD25 | *xzEx25[Pvha-6::Dendra2]* | [35] |
| [7]YD27 | *xzEx27[Pvha-6::UbG76V::Dendra2]* | [35] |
| [8]YD90 | *xzIs1[Pvha-6::UIM2::ZsProSensor]* | [28] |
| [9]YD116 | *rrf-3(pk1426);xzls2[Punc-54::UIM2::ZsProSensor]* | Generated for this study |

[1] Wild-type Bristol strain

[2] RNAi-sensitive strain

[3] Dual fluorescent reporter for autophagy studies

[4] and [6] Dendra2, a photoconvertible green-to-red fluorescent protein expressed in the body-wall muscle and intestinal cells, respectively.

[5] and [7] UbG76V-Dendra2, which consists of the non-hydrolyzable ubiquitin moiety UbG76V fuse to Dendra2. The photoconvertible UbG76V-Dendra2 is degraded by the proteasome and measures UPS-mediated protein degradation independently of translation of reporter proteins, expressed in the body-wall muscle and intestinal cells, respectively.

[8] and [9] Fluorescent polyubiquitin reporter, which binds to endogenous LYS-48-linked polyubiquitinated proteasomal substrates, expressed in the intestinal and muscle cells, respectively.

**Table S2.** **Numbers of imaged polyubiquitin reporter animals**

| Strain | Imaged tissue | RNAi | Number of animals imaged (number of experiments) | p-value against control RNAi |
|---|---|---|---|---|
| *YD90[xzIs1[Pvha-6::UIM2::ZsProSensor]]* | Intestine | *L4440* | 90 (6) | |
| | | *lgg-1* | 90 (6) | 0,010667 |
| | | *lgg-2* | 75 (5) | 0,020198 |
| | | *bec-1* | 90 (6) | 0,013207 |
| | | *atg-7* | 75 (5) | 0,007871 |
| | | *rab-7* | 90 (6) | 0,045469 |
| | | *epg-5* | 90 (6) | 0,833626 |
| | | *sqst-1* | 90 (6) | 0,141818 |
| | | *sqst-1+lgg-1* | 90 (6) | 0,034358 |
| *YD116[rrf-3(pk1426);xzls2[Punc-54::UIM2::ZsProSensor]]* | Body-wall muscle | *L4440* | 90 (6) | |
| | | *lgg-1* | 75 (5) | 0,055731 |
| | | *lgg-2* | 90 (6) | 0,009538 |
| | | *bec-1* | 75 (5) | 0,000847 |
| | | *atg-7* | 60 (4) | 0,411909 |
| | | *rab-7* | 60 (4) | 0,384942 |
| | | *epg-5* | 90 (6) | 0,000762 |

| | | | | |
|---|---|---|---|---|
| | | *sqst-1* | 50 (3) | 0,211944 |
| | | *sqst-1+epg-5* | 50 (3) | 0,055069 |

**Table S3. Numbers of imaged UbG76V-Dendra2 and Dendra2 reporter animals**

| Strain | Imaged tissue | RNAi | Number of animals imaged (number of experiments) | p-value against control RNAi (degradation UbG76V-Dendra2) |
|---|---|---|---|---|
| *YD27[xzEx27[Pvha-6::UbG76V::Dendra2]]* | Intestine | *L4440* | 115 (5) | |
| | | *lgg-1* | 112 (5) | 0,0016458 |
| | | *lgg-2* | 105 (5) | 0,0004006 |
| | | *bec-1* | 112 (5) | 0,0002728 |
| | | *atg-7* | 105 (5) | 0,00497 |
| | | *rab-7* | 100 (5) | 0,29413 |
| | | *epg-5* | 105 (5) | 0,1925351 |
| | | *sqst-1* | 65 (3) | 0,2417188 |
| | | *sqst-1+lgg-1* | 65 (3) | 0,0202486 |
| *YD25[xzEx25[Pvha-6::Dendra2]]* | Intestine | *L4440* | 65 (3) | |
| | | *lgg-1* | 65 (3) | 0,3441761 |
| | | *lgg-2* | 63 (3) | 0,1544679 |
| | | *bec-1* | 65 (3) | 0,0657256 |
| | | *atg-7* | 60 (3) | 0,286508 |
| | | *rab-7* | 65 (3) | 0,0978616 |

| | | | | |
|---|---|---|---|---|
| | | *epg-5* | 70 (3) | 0,1084852 |
| *YD3[xzEx3[Punc-54::UbG76V::Dendra2]]* | Body-wall muscle | *L4440* | 105 (5) | |
| | | *lgg-1* | 105 (5) | 0,917595 |
| | | *lgg-2* | 105 (5) | 0,145795 |
| | | *bec-1* | 105 (5) | 0,978631 |
| | | *atg-7* | 105 (5) | 0,735148 |
| | | *rab-7* | 105 (5) | 0,217689 |
| | | *epg-5* | 105 (5) | 0,032891 |
| *YD1[xzEx1[Punc-54::Dendra2]]* | Body-wall muscle | *L4440* | 65 (3) | |
| | | *lgg-1* | 65 (3) | 0,44017 |
| | | *lgg-2* | 60 (3) | 0,62759 |
| | | *bec-1* | 65 (3) | 0,422 |
| | | *atg-7* | 60 (3) | 0,95776 |
| | | *rab-7* | 65 (3) | 0,16752 |
| | | *epg-5* | 65 (3) | 0,82245 |

**Table S4. Significance values of *in vitro* assays**

| Approach | RNAi | Number of experiments | p-value compared to control RNAi | |
|---|---|---|---|---|
| *In-gel* proteasome activity assay | | | From whole animal lysates | |
| | *L4440* | 10 | | |
| | *lgg-1* | 10 | 0,008881 | |
| | *lgg-2* | 10 | 0,257055 | |
| | *bec-1* | 10 | 0,720861 | |
| | *atg-7* | 10 | 0,520055 | |
| | *rab-7* | 10 | 0,149359 | |
| | *epg-5* | 10 | 0,000215 | |
| Western Blotting | | | From whole animal lysates | |
| | *L4440* | 6 | | |
| | *lgg-1* | 6 | 0,643918 | |
| | *lgg-2* | 6 | 0,0006 | |
| | *bec-1* | 6 | 0,003655 | |
| | *atg-7* | 6 | 0,05713 | |
| | *rab-7* | 6 | 0,331061 | |
| | *epg-5* | 6 | 0,655201 | |
| Immunohistochemical analysis | | | Intestinal cells | Body-wall muscle cells |
| | *L4440* | 3 | | |
| | *lgg-1* | 1 | NA | NA |
| | *lgg-2* | 3 | 0,697217 | 0,035077 |
| | *bec-1* | 3 | 0,015465 | 0,010164 |

| | | | | |
|---|---|---|---|---|
| | *atg-7* | 1 | NA | NA |
| | *rab-7* | 1 | NA | NA |
| | *epg-5* | 3 | 0,34077 | 0,198936 |

**Table S5.** **Numbers of imaged *mCherry::GFP::LGG1* reporter animals**

| **Strain** | **Imaged cell** | **RNAi** | **Number of cells imaged (number of experiments)** | **p-value against control RNAi** |
|---|---|---|---|---|
| *MAH215[sqIs11[lgg-1p::mCherry::gfp::lgg-1 + rol-6]]* | Hypodermal seam cell | *L4440* | 15(3) | |
| | | *lgg-1* | 15(3) | 0,000816 |
| | | *lgg-2* | 15(3) | 0,000199 |
| | | *bec-1* | 15(3) | 0,062158 |
| | | *atg-7* | 15(3) | 0,000386 |
| | | *rab-7* | 15(3) | 4,01E-06 |
| | | *epg-5* | 15(3) | 0,000131 |

**Table S6. List of qPCR oligonucleotides used in this study**

| ***C. elegans*** | **Forward** | **Reverse** |
|---|---|---|
| *lgg-1* | aggagacaagatccgcagaa | gacgaagttggatgcgtttt |
| *lgg-2* | ttagacgacgcctccaactt | ctggatcacgctcttgactg |
| *bec-1* | gatcctgttggagcgtatcg | cgaattccaggatcaattcc |
| *atg-7* | atcgcttcatcaaaccgaag | agggtaccggacattgaca |
| *rab-7* | ttacgaggtttctgccaagg | ggaaatcgttggtttcctga |
| *epg-5* | ctccaccaccacgtgtttc | tggtgctaccgctgtagttg |
| *act-1* | tcggtatgggacagaaggac | catcccagttggtgacgata |
| *cdc-42* | ctgctggacaggaagattacg | ctcggacattctcgaatgaag |
| *pmp-3* | gttcccgtgttcatcactcat | acaccgtcgagaagctgtaga |
